# 50 Years Journal of Mathematical Biology

**DOI:** 10.1007/s00285-025-02259-0

**Published:** 2025-10-06

**Authors:** Thomas Hillen, Anna Marciniak-Czochra

**Affiliations:** 1 Department of Mathematical and Statistical Sciences, University of Alberta, Edmonton, Canada; 2 Institute of Applied Mathematics, University of Heidelberg, Heidelberg, Germany

## Abstract

The year 2024 marked the 50th anniversary of the Journal of Mathematical Biology. The journal was founded in 1974 with the vision to build a platform for advanced mathematical methods as they are applied and developed for biological problems. What began as a small journal for a specialized group of experts has grown into a flag-ship journal of a large and ever expanding field. We celebrate this occasion with a Special Collection of papers from our Associate Editors and our past and present Editors in Chief to showcase the state of the art and stimulate interesting new research directions in Mathematical Biology.

## The past and present

The Journal of Mathematical Biology was established in 1974 after a series of discussions between Axel Springer (founder of Springer Verlag) and KP. Hadeler of the University of Tübingen in Germany. It was founded under the leadership of KP. Hadeler, H. Bremerman, and F.A. Dodge. The editorship changed over the years, and in Table 1 we summarize the Editors in Chief and their terms in service. It should be noted that the Chief Editors were called “Managing Editors” in the 1970–1990s. The engagement of KP. Hadeler and S. Levin transformed the young journal into a self-assured and goal oriented platform. All Editors continued to build the journal into a leading venue of scientific exploration and we (Anna and Thomas) are extremely grateful for their leadership and dedication.

**Table 1:** Managing Editors and Editors in Chief of the Journal of Mathematical Biology

Hans Bremermann	Founding editor 1974–1979
Fred A. Dodge	Founding editor 1974–1979
Karl Peter Hadeler	Founding editor 1974–2004
Dezsö Varju	1976–1979
Simon A. Levin	1976–1995
Alan Hastings	1995–2008
Odo Diekmann	1998–2008
Mark A. Lewis	2008–2023
Mats Gyllenberg	2009–2022
Anna Marciniak-Czochra	Since 2021
Thomas Hillen	Since 2022

**Fig. 1:**
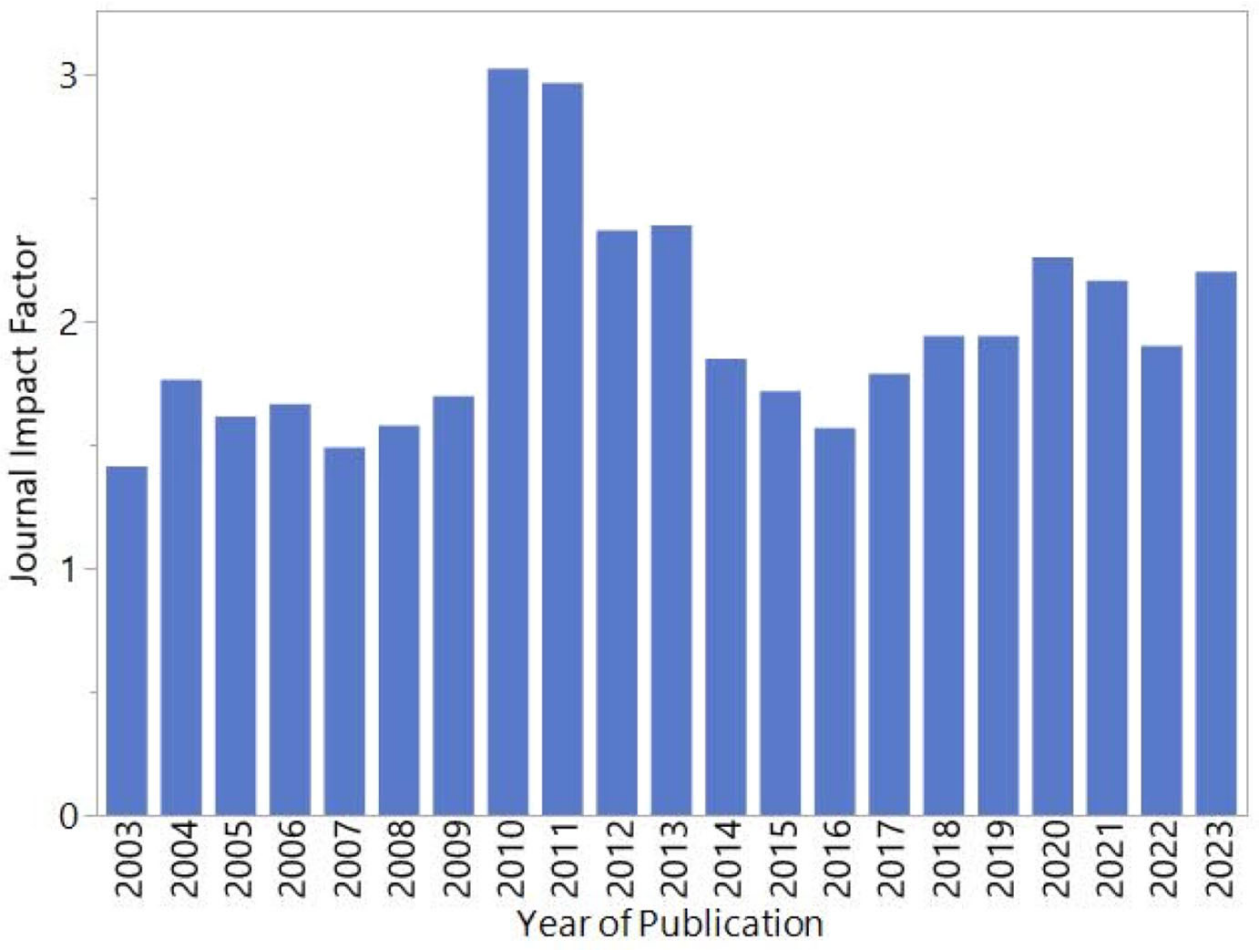
Journal Impact Factor since 2003. (Thanks to Lisa Hillen for this graphic)

No journal analysis is complete without a discussion of the ever so important impact factor. In Fig. 1 we present the rise and fall of our impact factor since 2003. Beginning at about 1.5 we climbed to 3.0 in 2010/11 and returned to about 2 in subsequent years. Last year we climbed from 1.9 to 2.2, and this year (2025) we reached 2.3. If you wonder if such a small increase is significant, we can tell you that the number of paper submissions increased by 70% this year.

To better understand the dynamics of the impact factor we look at the top ten cited papers of the journal in Table 2. The topics of these papers reflect the scope of the journal quite nicely, covering epidemiology, ecology, evolution, neuronal networks, spatial spread, tumor dynamics and a rather theoretical work on Poincare-Bendixson type of results for asymptotically autonomous differential equations in the plane. The indisputable leader is the paper by O. Diekmann, JAP. Heesterbeek, and JAJ. Metz, where the idea of a basic reproduction number $R_0$ is developed in a systematic way, and made available to the wider field of mathematical epidemiology. The paper by E. Oja on neuronal networks has become one of the foundational papers in AI and machine learning. The Users Guide on Chemotaxis by T. Hillen and KJ. Painter stands out as the youngest paper in this list. It came out in 2009 and it stimulated new research on chemotaxis type models and their applications. It became a major contributor to the increased impact factor in the following years and shows that well placed review articles can have a substantial impact on the field.

**Table 2:** Top ten cited papers of the JOMB. The citation numbers are taken from Google Scholar on April 15, 2025

Article	Citations
Diekmann, O., Heesterbeek, JAP. and Metz, JAJ. On the Definition and the Computation of the Basic Reproduction Ratio $R_0$ in Models for Infectious-Diseases in Heterogeneous Populations. 28(4) 365–382, 1990	6303
Oja, EA. Simplified Neuron Model as a Principal Component Analyzer, 15(3) 267–278, 1982	3499
Hillen, T. and Painter, KJ. A User’s Guide to PDE Models for Chemotaxis, 58(1–2) 183–217, 2009	1872
Dieckmann, U. and Law, R. The Dynamical Theory of Coevolution: A Derivation from Stochastic Ecological Processes, 34(5–6) 579–612. 1996	1395
Othmer, HG., Dunbar, SR. and Alt, W. Models of Dispersal in Biological Systems. 26(3) 263–298, 1988	1145
Kirschner, D. and Panetta, JC. Modeling Immunotherapy of the Tumor-Immune Interaction 37(3) , 235–252, 1998	1124
Liu, WM., Levin, SA. and Iwasa, Y. Influence of Nonlinear Incidence Rates upon the Behavior of SIRS Epidemiological Models. 23(2) 187–204, 1986	1083
Liu, WM., Hethcote, HW. and Levin, SA. Dynamic Behavior of Epidemiologic Models with Nonlinear Incidence Rates. 25(4), 359–380, 1987	1056
Thieme, HR. Convergence Results and a Poincare-Bendixson Trichotomy for Asymptotically Autonomous Differential Equations, 30(7), 755–763, 1992	894
Kuang, Y; Beretta, E Global Qualitative Analysis of a Ratio-Dependent Predator–Prey System 36(4) 389–406, 1998	774

In Table 3 we list the top ten authors of the journal. We recognize names of leaders in the field and several of them were, or currently are, Associate Editors or Editors in Chief. Two of the top authors are women, which is remarkable as we span the time from 1974 until now.

**Table 3:** Top ten authors of the JoMB and their number of papers as of December 2024

Name	Number of articles in JoMB
Philip Maini	32
Pauline Van Den Driessche	29
Johan A.J. Metz	28
Odo Diekmann	28
Helen M. Byrne	27
Mark A. Lewis	25
Mats Gyllenberg	24
Hao Wang	22
Hans G. Othmer	21
Shigui Ruan	21

In 2002 the JoMB became the house journal of the European Society for Mathematical and Theoretical Biology (ESMTB, https://www.esmtb.org). This collaboration has been very successful, leading to increased scope and readership. In 2022 the ESMTB and the Journal launched the KP. Hadeler Prize, in honor of one of its founders. It awards the best paper of a given year and the first three papers that won the KP. Hadeler Prize are:

- *2022:* I. Mazari and D. Ruiz-Balet. Spatial ecology, optimal control and game theoretical fishing problems. JoMB 85(55).
- *2023:* L. Kläy, L. Giardin, V. Calvez and F. Débarre. Pulled, pushed or failed: the demographic impact of a gene drive can change the nature of its spatial spread. JoMB 87(30).
- *2024:* M. Benaim, C. Lobry, T. Sari, E. Strickler. When can a population spreading across sink habitats persist? JoMB (2024) 88(19).

## The future

The mainstream research in biology, ecology, and medicine has developed into data generating machines. Data are collected on every conceivable scale, starting with genomics, proteomics, transcriptomics, single cell mRNA sequencing, cell tracking, medical imaging, tissue, organ and systemic data, as well as animal behavior, reproduction, species interactions, species land use, and millions of individual movement paths of animals. The necessity for robust data analysis tools is becoming increasingly apparent, with this demand driving the development of new approaches such as artificial intelligence, statistical learning, information theory and topological or geometric methods. The value of mathematical modelling has always been its capacity to identify structures, to comprehend complex mechanisms at a profound level, and to establish connections between disparate phenomena. This remains a fundamental strength in the present era. However, there is more to it than that. The advent of innovative data analysis tools has given rise to a new wave of mathematical challenges, which we see as a natural direction of growth for our journal.

Another challenge that JoMB, and essentially all journals face, is the systematic exploitation of publication venues for fraudulent papers, ghost authors, papers generated by AI, and paper mills. The extent of opportunities to purchase authorship on a paper is staggering. Publishers, such as Springer, work hard to filter out these papers before they go to the editors. This requires resources and state of the art AI tools. As long as the numbers of papers and the impact factor decide about someone’s career, the temptation of exploitation will not go away.

Some of our colleagues are quite concerned about the future of scientific publishing. They fear that the publication landscape will be so diluted by fake and fraudulent papers, that it becomes worthless. We are more optimistic. Advances in science will always be beneficial for society. Modern research is always based on research done previously and serious research labs will ensure to use sources they can trust. Science will find a way to distinguish good from bad, which will probably lead to an even more elite publication landscape. Journals of high reputation will flourish and become even more selective. Some few journals will dominate their fields. The JoMB has a very high reputation within our area, and it is our aim to continue the publication of high quality research in the future.

We thank all editors, authors, reviewers, and readers for their contributions to the success of the journal. Your contributions have made what the journal is today, and we are excited to continue the promotion of ground breaking research in Mathematical Biology. Our editorial team is ready to face whatever the future brings, to ensure the best papers find their way into the Journal of Mathematical Biology.

Anna Marciniak-Czochra (Heidelberg)

Thomas Hillen (Edmonton)

